# Data on bioassay of toxicity reduction of treated textile wastewater by using nanophotocatalytic process by *Daphnia magna*

**DOI:** 10.1016/j.dib.2018.10.143

**Published:** 2018-10-30

**Authors:** Marjan Ghanbarian, A.H. Mahvi, Maryam Ghanbarian

**Affiliations:** a School of Public Health, Shahroud University of Medical Sciences, Shahroud, Iran; b Center for Solid Waste Research, Institute for Environmental Research, Tehran University of Medical Sciences, Tehran, Iran; c Ministry of Health and Medical Education, Tehran, Iran

**Keywords:** Bioassay, *Daphnia magna*, Nanophotocatalytic, Titanium dioxide, Dye RO 16

## Abstract

Practicability and possibility of photocatalytic degradation of Ro16 textile dye and the actual wastewater of textile were studied on pilot scale. The amount of reduction in solution toxicity was studied and assessed by the application of a bioassay using *Daphnia magna*. The solution toxicity at the beginning of the process has an increasing procedure and this is caused by the intermediate products that are produced during the photocatalytic process from the mother compounds, and are more toxic compared to them, and their toxicity declines at the end of the process with the completion of mineralization. The procedure of toxicity increase and its decrease in the course of photocatalytic process has a direct relation with the amount of mineralization.

**Specifications table**

**Table:** 

Subject area	Environmental pollution
More specific subject area	Industrial wastewater monitoring
Type of data	Figure
How data was acquired	Taking samples and conducting the toxicological tests and analyzing the results of the data
Data format	Analyzed
Experimental factors	Detoxification of industrial wastewater and mortality of *Daphnia* in different time intervals
Experimental features	Upon sampling and analyzing the obtained data, the comparative results of *Daphnia* mortality are shown in the figures, and the toxicity is evaluated.
Data source location	*Baft Azadi* textile factory and the laboratory
Data accessibility	Data are available in the article
Related Research	A.H. Mahvi, A. Maleki, M. Alimohammadi, A. Ghasri,
	Photo-oxidation of phenol in aqueous solution: Toxicity of intermediates, Korean Journal of Chemical Engineering, 24 (2007) 79–82

**Value of the data**

• Detoxification of textile wastewater from reactive dyes using photocatalytic processes can help to have better quality of treated wastewater for agricultural, breeding fish or feeding groundwater.
• Mortality rate of *Daphnia* indicated whether detoxification of textile wastewater is successful or not?
• Monitoring of the treated wastewater is one of the most important applied and practical aspects of the procedure.

## 1 Data

**Fig. 1:**
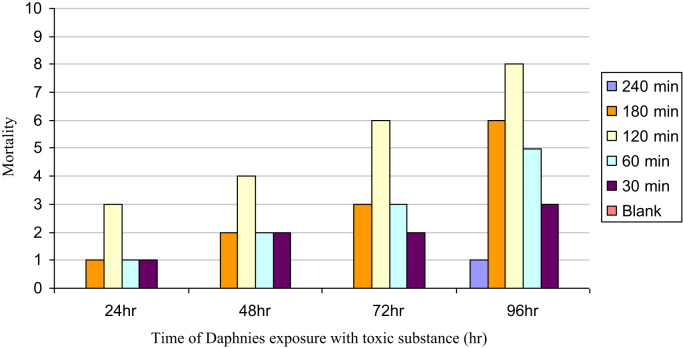
Daphnia mortality rate in synthetic solution RO 16.

**Fig. 2:**
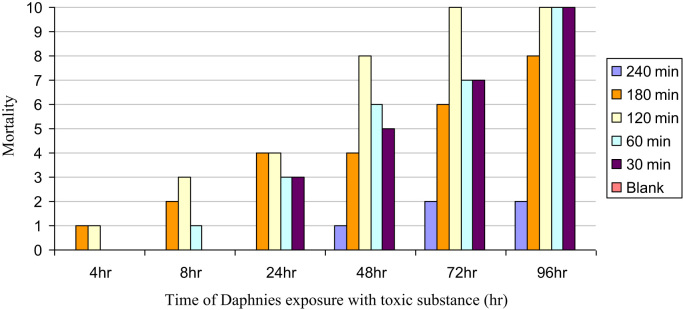
Daphnia mortality rate in real textile wastewater sample No.1.

**Fig. 3:**
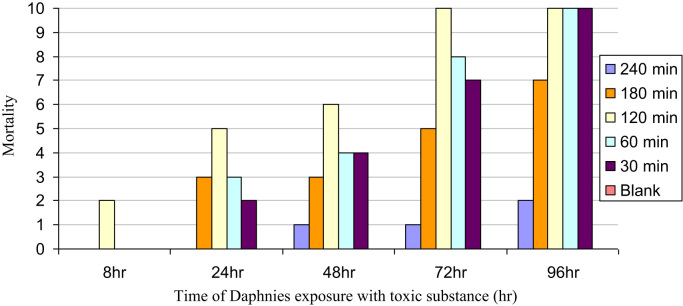
Daphnia mortality rate in real textile wastewater sample No.2.

Nowadays, among the methods of toxicity monitoring and tracing, bioassay with *Daphnia*, as a result of its specific characteristics, is one of the most common methods. The purpose of this experiment was the specification of toxicity reduction of synthetic solution of RO 16 dye and 2 real textile wastewater samples at different times in the course of photocatalytic reaction by TiO_2_. Synthetic solutions of RO 16 dye and real "*Bafte Azadi*" textile wastewater were treated by photocatalytic process using nano-particles of TiO_2_ in a reactor. After treatment process, the nanoparticles of TiO_2_ were separated by filtering after centrifuge in 6000 rpm for each sample. The treated samples were exposed to toxicity tests. In each sample tube and each control tube, 10 daphnia infants were placed. The concentration of the toxic substance in the control cell was 0. The monitoring and evaluation of the sample tube contained *Daphnia*, were performed regularly and accurately after 24, 48, 72, 96 hours and also during different irradiation time of 30, 60, 120, 180 and 240 min. The number of stagnant daphnia was recorded in each experiment. All the tests were done with 10 live *Daphnia*. The results of this monitoring and evaluation are shown in Fig. 1, Fig. 2, Fig. 3. Vector y represents the number of dead *Daphnia* and vector x represents time of *Daphnia* exposure to treated wastewater which is grab in different photocatalytic process time.

Nowadays, there are a few textile wastewater treatment processes with logical efficiencies in use by industries [1], [2], [3], [4], [5], [6], [7], [8], [9], [10], [11], [12], [13]. One of the processes which is able to deal with the degradation problems of dyes in aqueous solutions is Advanced Oxidation Processes (AOPs). AOPs react on the base of the production of very active hydroxyl radicals OH• which oxidize a vast range of pollutants fast and non-selectively. Among AOPs, it seems that the heterogeneous photo catalytic process using TiO_2_ as a catalyst is a more destructive technology [14], [15], [16]. RO 16 has a maximum absorption in 497 nm visible area. It is evident that the conduction band electrons (e^-^) and the valence band holes (h^+^) are formed when the aqueous suspension of TiO_2_ irradiates with an energy lower than its own band-gap energy (3.2 ev.Eg) [1], [17].

## 2 Experimental design, materials and methods

### 2.1 Reagent

In this paper, Reactive Orange 16 was provided by "*Alvan Sabet*" company in Iran. Reactive Orange 16 (C.I. 17757) (R3R) is a reactive dye bearing an azo group as chromophore and a sulphato-ethylsulfone as the reactive group. Titanium dioxide (Degussa P25) was utilized as a photocatalyst. Its main physical data are as follow: average primary particle size around 21 nm, purity ~ 99.5% and BET surface area 50 ± 15 m^2^ g^-1^. A *UV-C* 18 W lamp (Philips) was used as irradiation source.

### 2.2 Photocatalytic reactor

Experiments were carried out in a batch mode immersion rectangular photocatalytic reactor made of glass. An 18 W low pressure mercury lamp was placed in the center of the photocatalytic reactor as the UV irradiation source that protected by quartz jacket. The slurry composed of dye solution and catalyst placed in the reactor was placed on a magnetic stirrer and stirred magnetically. Samples after photocatalytic treatment were centrifuged (6000 rpm, 10 min) and were filtered through Millipore filter (0.45 µm) membrane. Photocatalytic degradation processes were performed using a 2.3 L solution containing specified concentration of selected dye. Samples were withdrawn from sample point at certain time intervals and analyzed for detoxification rate by using *Daphnia* bioassay.
