# Supplementary material for: Data on bioassay of toxicity reduction of treated textile wastewater by using nanophotocatalytic process by Daphnia magna
Source: Data Brief. 2018 Oct 30;21:1321–4. doi: 10.1016/j.dib.2018.10.143 (PMC6231081; doi:10.1016/j.dib.2018.10.143)
Supplement: Supplementary file 1 — Supplementary material [file mmc1.docx]

Dear Editor

There is no conflict of interest in this paper.

Thanks

Mahvi
